# Hospital and Institutional News

**Published:** 1912-11-23

**Authors:** 


### HOSPITAL AND INSTITUTIONAL NEWS.

### THE DOCTORS' DECISION.

A week or more ago it had become plainly apparent that the general feeling among the divisions and branches of the British Medical Association was strongly adverse to acceptance of the Chancellor's revised terms, which were so enthusiastically received and recommended by Sir Clifford Allbutt. That anticipation of refusal was confirmed on Tuesday at the meeting of representatives of the Association, and the refusal plunges Mr. George and his Act into a worse dilemma than ever. By Fabian tactics he managed to postpone for fifteen months that settlement with the profession which ought to have been arrived at before the Bill was ever passed at all. Whether by this delay he thought to wear down opposition or to reconcile it, it is idle to speculate on now. Suffice it that he has done neither, and that now, less than two months before medical benefit is to begin, he seems as far as ever from being able to provide it. The decision of the representatives against service on the terms lately proposed was almost unanimous. But negotiations are not broken off, for a further resolution was passed by a small but sufficient majority empowering the Association to negotiate for the improvement of the conditions of service. This course has been advocated in these columns with the hope that the ablest negotiators may be chosen. We congratulate the Association on its adoption, and hope that the result of further negotiation with the Chancellor will be more satisfactory.

### THE MENTAL DEFICIENCY BILL DROPPED.

As was not unexpected, in view of the situation created by the defeat of the Government last week, the Home Secretary announced at the meeting of the Standing Committee which has been considering the Mental Deficiency Bill that the Government could no longer be bound by their promise to endeavour to pass it into law this session. He went on to say that the Government hoped to make the Bill, as amended in Committee, the basis of a similar measure next session. Mr. McKenna then asked the Committee to attend two or three more meetings so that they might help him to get rid of Clauses 17 and 12, and one or two others that are likely to be the cause of controversy.

### THE INSURANCE ACT AND RESIDENT STAFFS: EXEMPTION DECIDED ON.

The Insurance Commissioners have decided that resident medical officers are not employed under a contract of service within the meaning of the National Insurance Act, and consequently are not required to be insured, even if their rate of remuneration does not exceed in value £160 a year. This decision, it will be remembered, is the outcome of the application made to the Insurance Commissioners, which led to a public hearing being held on October 22 last. This decision is one of great importance, and will be welcomed by the hospital authorities throughout the country. It may, indeed, be even more important than it sounds, for the recognition of the principle that one class of hospital workers is to be exempted may not have an unfavourable influence now that other sections of the hospital world, notably the nurses, are pressing their claims.

### THE NEW ST. ANDREW'S HOSPITAL.

A new institution, to be known as St. Andrew's Hospital, is being built at Dollis Hill, Willesden, and it is expected will be open for the reception of patients in January next. The foundress is an anonymous lady, who has placed in the hands of Cardinal Bourne sufficient funds to carry on the hospital, to which French-speaking Roman Catholics will have priority, but Protestants eligibility, of admission. The objects of the hospital are stated in addition to include the admission of patients who do not care to receive free treatment, but who can afford only small fees. The accommodation will consist of one hundred beds for medical and surgical but not for infectious or mental cases. The hospital will be staffed and administered by the Poor Servants of the Mother of God, some of whom, we understand, have been trained at the London and at St. George's Hospital. As the name of the site implies, the hospital building, it is claimed, "occupies a high and open position," which the growth of London is understood to be incapable of affecting. Its appearance is cheerful, the elevation being of red brick and Portland stone dressings. The central block has a stone façade surmounted by a dome, while at each corner there is a flanking tower containing the sanitary annexes. The building has four floors, with a terrace at the ground and a balcony at the first-floor level. The front garden is being laid out in lawns, and the kitchen garden will be placed behind the building. Mr. Robert L. Curtis is the architect. It should be added that there is already a Roman Catholic Hospital, that of St. John and St. Elizabeth, at St. John's Wood, and the question of consolidating it was taken into consideration before it was decided to erect a new building. It is, we gather from the papers sent us, a Home rather than a Hospital. It will also be noticed that the Willesden site does not follow the prevailing fashion of new London hospitals, in that it is in the north and not in the south of the Metropolis.

### A MENTAL PATIENT CHARGED WITH MURDER.

The authorities of Long Grove Mental Hospital have had the misfortune to have one of their patients committed for trial at the Epsom Police Court on the charge of murdering another patient in the institution. The accused is named Jim Mitchell, aged thirty-seven, a coloured man, who has been a patient at Long Grove for some four years, during which time he has worked beside the man with whose murder he is now charged. The two men, it appeared, were at work in a cowshed when the prisoner came up to a farm attendant saying, "I have hit him with a broom," and then went on with his work quite calmly. The farm attendant said that he found the dead man lying with his head battered in and a broken broom beside him. The witness added that the prisoner had been very quiet during his previous time in the hospital. In giving evidence, Dr. S. F. Barham stated that the prisoner remarked to him that there had been a fight; and that he was regarded as quite harmless. Dr. Barham further explained that the prisoner suffered originally from delusions, thinking that he was the Apostle Peter, and also that he was being constantly persecuted. Dr. Mapother, assistant medical officer at Long Grove, did not think that the prisoner understood the proceedings. All hospital workers will sympathise with Dr. Barham and the staff at Long Grove at the present juncture, which illustrates the peculiar responsibilities and anxieties attaching to mental hospital work.

### SECRET REMEDIES—THE CASE FOR THE PROPRIETORS.

The second stage of the inquiry into the Sale of Proprietary Remedies began at the sitting of the Select Committee, at which Mr. John C. Umney, representing among other interests the "Owners of Proprietary Articles Section" of the London Chamber of Commerce, gave evidence. He said that the Section had given the most careful consideration to the suggestions put forward by Dr. Cox on behalf of the British Medical Association, and that in the opinion of the members the disclosure of formula would enable others to make the article and describe it as made according to the original formula. This would be the means of transferring a large portion of trade from the original inventor to other competitors. He assured the Committee that any proposed amendment of the law which would compel owners of proprietary articles to disclose their trade secrets would be strongly opposed, but that, on the other hand, the Section would welcome legislation embodying the following principles:—(*a*) As regards abortifacients, anti-conception remedies, and remedies purporting to affect sexual virility, there might be stringent restrictions forbidding newspaper or circular advertisement. (*b*) As regards remedies advertised as specially applicable to ailments peculiar to women, the public advertisement should be in simple language such as not to imply that the medicine was recommended for improper purposes. (*c*) As to remedies claiming to cure the diseases of cancer, consumption, rupture (other than by mechanical appliances), locomotor ataxy, Bright's disease, diabetes, etc., public newspaper or circular advertisement of any such medicines might be made illegal, and no statement should be made on any label on any medicine that it would cure any of such diseases.

### MOTHER SEIGEL'S ORIGIN.

The Inquiry into the Sale of Proprietary Medicines reached an interesting stage at the last meeting of the Select Committee, when Mr. C. H. Radcliffe, a director of A. J. White, Ltd., proprietors of Seigel's Syrup, appeared to give evidence. The founder of the business gave great publicity to the statement that there was a certain old woman, "Mother Seigel," who, wandering about the lanes of her native village in Germany one day, plucked a herb which gave her immediate relief from the disease from which she was suffering. The Chairman asked Mr. Radcliffe whether this statement was false, and, after admitting that it was purely fanciful, the witness was further pressed by the Chairman and said, "I am prepared to say that in my opinion it was a false statement, but my opinion counts for little, because I have never attempted to satisfy myself about it one way or the other." Questioned about the composition of the preparation, Mr. Radcliffe said it contained eleven extracts, and that the statement published in "Secret Remedies" was grossly misleading; he was not at liberty, however, to state what these extracts were. During the sitting the Chairman made a statement regarding a matter which had arisen in the course of the sittings—namely, the action taken by the City of Liverpool concerning the sale of certain medicines. The Committee had communicated with the Liverpool authorities, and had received the following letter from Dr. E. W. Hope, the medical officer of health:—"In accordance with your request for information as to the procedure adopted by the Health Committee of the Corporation of Liverpool, I beg to state that in the year 1907 the Health Committee asked for information relating to the sale of proprietary medicines, and an analysis was made of a number of such articles. In connection with one of them—namely, Guy's Tonic—information was laid against the vendors on the grounds that the printed advertisements state that 'Guy's Tonic' contains the active ingredients and digestive qualities of the gastric juices in a highly concentrated and permanent form." The price of the bottle was 2s. 9d., and the value of the contents would appear to be about a penny. The case was withdrawn, as no evidence of guilty knowledge on the part of the vendor could be brought forward, and the stipendiary magistrate allowed the defendants five guineas costs. The Chairman read from the analyst's certificate as follows: "I am of opinion that the said sample consists of water and 0.07 per cent. of phosphoric acid, 0.06 of hydrochloric acid, and 0.06 of nitric acid, with smaller doses of calcium phosphate, flavoured with chloroform, and coloured with cochineal. Except for the trace of hydrochloric acid, the sample is devoid of active ingredients of gastric juice, and has no digestive properties whatever."

### DEATH OF A HOSPITAL ARCHITECT.

We regret to announce the death of Mr. Edward Blakeway I'Anson, the well-known architect, at the age of sixty-nine years. The son of an eminent architect, he took his degree from St. John's, Cambridge, and became a Fellow of the Architects' Institute in 1880, and was elected for this year (1912-1913) as Vice-President of the Surveyors' Institution. Probably Mr. I'Anson will best be remembered as Surveyor to the Governors of St. Bartholomew's Hospital. In 1892 he carried out some extensive sanitary improvements to the hospital buildings, and later (in conjunction with Mr. Rowland Plumbe, F.R.I.B.A.) prepared the design for rebuilding, estimated to cost £300,000. Among many other works he was also architect for Lord Alverstone's Cottage Hospital, Shanklin, Isle of Wight, and the Craig-y-don Convalescent Home, Llandudno.

### MR. WALTER ALVEY'S WARNING.

More than usual interest attaches to the annual dinner of the Incorporated Association of Hospital Officers which took place last Saturday. Mr. Stewart Johnson presided, and drew attention to the anxiety caused to hospital officials by the two subjects of medical inspection and the Insurance Act. In proposing the toast of the visitors, to which Lord Mersey, whose name has been before the hospital world as Chairman of the Special Committee on Out-patients, replied, Mr. Walter Alvey emphasised the need for common action by hospital authorities in view of the fact that the Insurance Act comes into force within two months' time. He said that he believed there was not a man who knew what the policy of the voluntary hospitals in relation to the National Insurance Act was going to be. If the voluntary system failed, Mr. Alvey proceeded, it would fail because those who were now responsible for hospital administration would not or could not understand that a united policy was essential. We are glad to report these words, because they echo the warning which The Hospital has repeated week after week for a long time past. If no one knows, as Mr. Alvey suggests, what the policy of the voluntary hospitals is going to be, no one, we confidently add, can fail to know what it ought to be who has been a consistent reader of this paper. In issue after issue we have laid down that the policy to be followed is one of *pro rata* payment for insured persons if the voluntary system is to remain intact, and we have illustrated this contention by reference to and comparison with the German system, while at the same time examining the provisions of the Insurance Act in a special department of the paper every week. No one has attempted seriously to dispute the solution we have proffered, and our last issue, dated as it happens on the same day as Mr. Alvey made his complaint of general apathy, opened with the warning: "Wake up, sleepers!" Will Mr. Alvey add to the value of his warning by explaining in the face of all that has been done what is the cause of the present inaction?

### SALARIES OF ASSISTANT MEDICAL OFFICERS.

As reported in The Hospital a fortnight ago, the Southwark Board of Guardians have considered the question of the salary paid to the third assistant medical officer at their infirmary, and, after comparing the amount of £120 a year with the salaries paid by similar bodies to the same officers, have decided that the sum is too small. The infirmary committee, in their report, state that £120 per annum, with the usual emoluments, is insufficient to attract highly qualified candidates to this position, and on their recommendation the Board unanimously decided to ask for the consent of the Local Government Board to increase it to £130. If approved the amount is to apply to the present holder of the office. The £10 rise is very small, but is a step in the right direction, and, it is urged, is more likely to be sanctioned by the Local Government Board than if a larger amount were proposed.

### ARCHITECTS PROTEST AGAINST AN AWARD.

The decision to extend the Menston Isolation Hospital, near Leeds, has involved the hospital committee in a protest from the competing architects. It appears that originally four firms were invited to submit drawings for a scheme to be carried out at a cost of £3,000, among the conditions attached being one that the designs should not be coloured, except on the block plan, and another that they should be unsigned. Among the competitors was Mr. Alfred Marshall, of Otley, who submitted two designs: one showing how the extension could be carried out for the sum stipulated, and a further set showing how, in his opinion, it ought to be carried out. The latter scheme, on the whole, impressed the hospital committee so favourably that with a few alterations it was accepted, at an estimated cost of £3,750. This decision has led to a protest from the remaining three competing firms, which allege that as the requirements have now been altered there should be a fresh competition. They also allege that the condition laid down as to the colouring of the plans was violated, and demand that all the competitive designs should be placed on exhibition, so that the public may decide as to their respective merits. The question really is: Should a hospital committee be bound by the strict conditions it has laid down in deciding on the plans sent in, or is it free to accept what it considers the best plans submitted, even though these violate the conditions laid down? What are our readers' opinions?

### A BEQUEST OF £6,000 REFUSED.

For the second time this year we have to record the refusal by a hospital of a bequest. The first instance occurred in February last, when the Manchester Eye Hospital declined a legacy of £130 because of the testator's condition that within twelve months two lady members should be appointed to the board of management. In the present instance the late Mr. John Jowitt, an estate agent, whose net personalty was valued at £9,422, bequeathed the residue of his estate, subject to certain legacies and to one life interest, to the Royal Lancaster Infirmary, provided that certain drastic conditions were fulfilled. These included stipulations that the infirmary should never receive State aid, and that the area from which patients might be received should be restricted. In these circumstances the committee of the Royal Lancaster Infirmary has decided that the bequest cannot be accepted, a weighty decision in view of the fact that the amount of the legacy was estimated at some £6,000. The reasons which led the committee to decide that in this case a bird in the hand was not worth two in the bush are so obvious that we need not insist upon them here. The life of the institution was clearly threatened by the testator's provisions. But in the present, as in the previous case, it is interesting to note that the cause of refusal can be found in the attempts of testators to affect the constitution of the hospitals which they set out to benefit.

### A SUCCESSFUL CANDIDATE DECLINES AN APPOINTMENT.

An unusual circumstance attended the recent election of Mr. C. J. Alexander, M.B., B.Ch., to the post of resident medical officer at Winsley Sanatorium, which serves the counties of Gloucester, Somerset, and Wiltshire. As a result of the voting of the board Mr. Alexander was placed second, the successful candidate being Mr. J. A. Kilpatrick, M.R.C.S. On being informed of his election Mr. Kilpatrick immediately declined the post on the ground that he had now discovered that the present medical locum at Winsley was a rival candidate for the position. Though learning that his action in declining the post would not cause the present locum tenens to be elected Mr. Kilpatrick replied that the fact would not alter his decision as he had very strong views on these matters. The board, including its medical members, regarded Mr. Kilpatrick's action as quixotic, and could see no reason why he should not accept the appointment. Mr. Alexander was then informed of the circumstances and accepted the committee's offer of the post. Mr. Alexander at present is resident medical officer to the Forster Green Sanatorium, Belfast. The successful medical candidate's quixotic action gave rise to varied comments, which included the suggestion that he was actuated by masonic motives. We feel, however, that no form of masonic or medical etiquette is capable of explaining what looks so like a personal freak of conduct.

### THE IMPORTUNATE CORONER.

No opportunity is lost at the City Coroner's Court of driving home both the abuses which certain obsolete enactments inflict upon the medical profession and the evil done by the want of repeatedly demanded legislation in other respects. Of the former the denial of fees to medical witnesses in Coroners' Courts is, perhaps, the most glaring instance, and Dr. Waldo, the City Coroner, is tireless in season and out of season in exposing the injustice caused by the existing law. Only the other day the payment of fees to two medical men on the staff of St. Bartholomew's Hospital for attendance was raised. The Coroner, in going over the old ground, remarked that he had never been able to obtain from anyone a defence of the provision in the Coroners' law whereby a medical man, if attached to a charitable institution, could be made to perform a public duty—namely, to give evidence at the inquest—without fee, while all other doctors were paid for it. The Departmental Committee *re* Coroners, he recalled, had reported: "We think that this inequitable provision of the Coroners Act of 1887 ought to be repealed, and, whatever may have been the original motives of policy for placing officers in a separate class and debarring them from receiving the fees payable to any other medical practitioners, we cannot see any reason whatever at the present time for continuing to deprive them of fees." He then repeated his suggestion that members of medical staffs should approach their members of Parliament and interview Mr. McKenna in order to bring about a reform. We heartily endorse this suggestion, and hope that his importunity may receive the success accorded to its most famous exemplar. It is really time that hospital staffs combined to register their protest in this matter.

### NEW BLOOD AT CHARING CROSS HOSPITAL.

The appointment of a new resident medical officer at Charing Cross Hospital marks a new departure for that institution, inasmuch as it is a break in the traditional policy of appointing to the office only men who have been trained in the medical school of the hospital—a policy which is followed by several other of the large London hospitals. There is much to be said for and against the principle that underlies such traditional policy, but we venture to think that most hospital authorities will agree that the introduction of new blood, provided it be good and not inferior to the traditional standard, is an innovation to be welcomed. In the case of one London hospital, at least, such an innovation has been the means of considerably raising the prestige and efficiency of the institution. The gentleman who will shortly take up this responsible post at Charing Cross Hospital is Mr. Bertram A. Lloyd, F.R.C.S., M.B., B.S. He has held the offices of house surgeon and house physician at the Queen's Hospital, Birmingham, and has also been house surgeon at the Hospital for Sick Children, Great Ormond Street.

### A CONDITIONAL GIFT AND ITS SEQUEL.

The Worthing Hospital has been placed in a curious position by the following circumstance. An appeal recently issued by the Mayor, Alderman Patching, for funds to pay off the debt on the hospital's new out-patient department, which has been built as the town's memorial to King Edward, was enforced by the statement that he had received a promise from an anonymous friend to give a sum equivalent to double the amount subscribed up to £500. To gain this £500 under the conditions imposed £250 had to be raised. Of this sum all but £20 was subscribed when the anonymous friend suddenly died. The question therefore arose, Was the hospital entitled or not to retain the contributions received in response to the appeal? It was eventually decided, with the approval of the hospital committee, that the Mayor should communicate with the contributors and offer to return their gifts because the conditions under which they were asked no longer obtained. Nor does the interest of the matter end here, for the story, like so many other hospital stories, has an unexpectedly happy ending. A legacy of £2,000 to the hospital has since been announced, and it is no longer a secret that the giver of the legacy and the anonymous friend were one and the same—namely, the late Alderman Cortis, an old friend of the institution and a member of its committee of management. Mr. Cortis has thus fulfilled the spirit if not a narrow reading of the letter of his promise, and it is hoped that all those who subscribed to the appeal will follow the example already set by some of the principal contributors, and not request the return of their money. Promises of large sums to hospitals contingent on the raising of similar or certain other amounts are nowadays such a popular way of increasing subscriptions, and, it might practically be said, have never been unfulfilled, that the present instance is worth recording, if only to remind hospital boards of the possibility of such a contingency.

### SECRETARY AND THREE TIMES MAYOR.

Mr. W. T. Patrick, J.P., F.C.I.S., Secretary of the Royal Surrey County Hospital, Guildford, till this year was one of that small but growing band of hospital secretaries who were also Mayors of their local towns. This year, however, he has, we believe, beaten all records in this connection by achieving re-election for the third year in succession to the office of Mayor of the county town. There is no need on this occasion to repeat our previous arguments as to the help it has generally proved to hospitals when the high civic offices in the towns are held by men conversant from practical experience with hospital work. The heavy calls on the time of hospital secretaries and on that of Mayors usually, however, are enough to preclude the same man from occupying the two posts. Mr. Patrick, however, appears to have solved their respective claims to the satisfaction of both hospital and town, for which feat he certainly deserves the prestige attaching to the record which his re-election has, apparently, established.

### THE ROYAL AUSTRALIAN NAVY.

In Monday's *Times* appeared an advertisement, inserted by the High Commissioner for the Australian Commonwealth, intimating that candidates are required for two appointments as surgeons to the Royal Australian Navy. From the wording of the notice it would not appear that the appointment carries with it his Majesty's commission; and indeed the appointment in the first instance is to be for a period of three years only, subject to re-engagement for a further period of three years. Nor are any details given of the emoluments, duties, privileges, and other conditions of the appointments; these can be obtained from the High Commissioner's office at 72 Victoria Street, S.W., to whom applications must be addressed not later than November 30. The peculiar significance of this announcement is, perhaps, more Imperial than institutional; but there must be many young hospital residents in Britain to whom the prospects of a three years' tour of service afloat in Australian ships of war will appeal very strongly. From the point of view of the consolidation of the Empire and its protection against foreign aggression, the creation of Colonial navies is a matter for congratulation.

### THE BIRMINGHAM HOSPITAL FOR WOMEN.

We have previously drawn attention to the work which the Birmingham and Midland Hospital for Women is doing in extending its accommodation for the reception of septic cases, which, till the institution opened its doors to patients suffering from puerperal fever, had virtually no beds in the city. In April 1911, it will be recalled, two beds were set aside for puerperal cases by arrangement between the hospital and the City Health Committee. This number needed soon to be raised to seven, and the demand has grown until arrangements have been made to open fourteen beds for this class of patient, three-fifths of the annual cost of which will be borne by the City Council. So far ten beds have been set aside exclusively for the work, and the additional beds bring the accommodation of the septic ward up to twenty-five. The result is that an appeal, strongly backed by Dr. John Robertson, the medical officer of health, has been issued for the extension of the hospital at Sparkhill. An operation room has been already built, but the extension will demand an increase of ten beds in the nurses' home. The sum asked for is £6,000, and the success which the institution of beds for this disease has already excited should promise well for the speedy raising of the money.

### VEXATIONS OF THE MOTOR SHOW.

Under existing arrangements visitors, especially those without technical knowledge, see mainly people, mostly males, rather than motors at the Motor Show. This must be ended or mended before next year's exhibition. Why not devote so many days for buyers and experts and so many for the general public? The most interesting place for non-technical persons to visit, if they had any powers of imagination, must undoubtedly have been the galleries, which were confined mostly to exhibitors who manufacture some of the innumerable accessories for which the motor industry has created a demand. It would be very interesting to know how many new trades and branches of manufacture this industry has called into being. We take it that for the younger generation who take an interest in machinery and mechanics the galleries at Olympia and the exhibits they contained must have had a supreme fascination. We would venture to suggest to the managers that it would be highly popular another year to arrange for the continual running of a cinematograph theatre in connection with the exhibition, where the whole mechanism of a motor-car could be shown and explained, and the differences between the various types of machines could be demonstrated. The doctor buying his first car would not fail to appreciate such an innovation.

### THE NEW WARDS AND THEATRE AT GREENWICH.

The new wing of the Miller General Hospital for South-East London, Greenwich, which, as we stated last week, was opened by Princess Louise, Duchess of Argyll, has certain points worthy of additional attention. First of all, the effect of the extension is to increase the number of beds from twenty-five to seventy-four, so that the size of the institution is practically trebled. The two round wards in the old building will probably be devoted to male medical cases and children respectively. One or two points require special notice. The electric light is reflected, and the windows are of that new patented pattern, which, as already illustrated in The Hopital, are divided into two, the top opening outwards in such a way that draughts are hindered and cleaning is greatly facilitated. Central stoves and radiators combine to heat the new wards. All the woodwork is of teak. The lockers are ingeniously devised in such a way that at meal times they can be moved alongside the bed, and provide a projecting table. The ward floors are of terrano, and each ward has a balcony wide enough to take the beds at right angles to the inner wall. Every ward kitchen possesses a ventilated store cupboard, and the kitchen trolleys are heated by electricity. The theatre unit includes among the customary rooms two new ones—namely, a bath room for the surgeons, and a changing room for the nurses.

### THIS WEEK'S DRUG MARKET.

Quiet conditions continue in the Drug market. Opium and its derivatives remain practically unaltered in price, and it is somewhat difficult to find a reason for the absence of excitement in this market, for an advance in the price of opium—possibly a substantial advance—seems almost inevitable. There has been no change in the prices of glycerine or cocaine since the advances announced last week. The rise in the value of menthol continues, and saffron is again dearer. At the recent public auction of drugs in Mincing Lane, Cape aloes tended lower in price, balsam tolu and sarsaparilla were cheaper, eucalyptus oil was dearer, and ipecacuanha tended lower.

### TO OUR READERS.

**Contributions are specially invited from any of our readers to these columns. They should deal with topical subjects and news. They must be authenticated for the information of the Editor only. The minimum payment if published is 5s. There is no hard-and-fast rule as to space, but notes of about twenty lines in length are preferred.**

